# Methodological assessment of the reduction of dissemination risk and quantification of debris dispersion during dissection with a surgical aspirator

**DOI:** 10.1186/s13104-022-05947-y

**Published:** 2022-03-02

**Authors:** Sosuke Kageyama, Atsuhiro Nakagawa, Tomohiro Kawaguchi, Kiyonobu Ohtani, Toshiki Endo, Manabu Kyan, Tetsuya Kusunoki, Yoshiteru Shimoda, Shin-Ichiro Osawa, Masayuki Kanamori, Niizuma Kuniyasu, Teiji Tominaga

**Affiliations:** 1 Department of Neurosurgery, Tohoku University Graduate School of Medicine, 1-1, Seiryo-machi, Aoba-ku, Sendai, Miyagi 980-8574 Japan; 2 Department of Biodesign, Clinical Research, Innovation, Education Center, Tohoku University Hospital, Sendai, Miyagi Japan; 3 Department of Neurosurgery, Kohnan Hospital, Sendai, Miyagi Japan; 4 Institute of Fluid Science, Tohoku University, Sendai, Miyagi Japan; 5 Department of Neurosurgical Engineering and Translational Neuroscience, Tohoku University Graduate School of Medicine, Sendai, Miyagi Japan

**Keywords:** Dissemination risk, Debris dispersion, Pulsed water jet, Optical absorbance, Evaluation method

## Abstract

**Objective:**

We developed an actuator-driven pulsed water jet (ADPJ) device to achieve maximal lesion dissection with minimal risk of normal structural damage. Despite the unique dissection characteristics, there is a risk of dissemination of tissue dispersion; however, there is no established method to quantify the dispersion. Hence, this study aimed to assess the factors associated with dispersion and propose a simple experimental method using spectrophotometry to evaluate the degree of dispersion in a wet field.

**Results:**

Methylene blue-stained brain phantom gelatin was immersed in a chamber with distilled water solution and dissected with an ADPJ. The dispersed gelatin solution was stirred and warmed to dissolve. The absorbance of the solution was measured spectrophotometrically. First, a reference standard curve was constructed to confirm the relationship between the absorbance and the amount of the dispersed gelatin. A clear proportional correlation was observed, which indicated that absorbance measurements can help evaluate the amount of dispersion. Using this method, we revealed that a high dissection force, insufficient suction, and inappropriate long distance between the nozzle tip and the target were associated with increased dispersion. This method might constitute a versatile and reliable approach to evaluate dispersion and aid in the development of surgical devices.

## Introduction

Maximal resection of the target lesion without causing critical damage to the surrounding normal structures is one of the most important goals of surgical treatment in the field of neurosurgery. Removal of deep-seated lesions, such as intraventricular tumors, is the most challenging procedure owing to the deep location and proximity to the eloquent area [1, 2]. Neuroendoscopy is an emerging technology that holds promise because it causes minimal stress to the approach corridor. However, it is not easy to remove ventricle lesions with a flexible neuroendoscope alone because of restrictions in the instruments that can be applied simultaneously through the working channel [1–5]. New devices that can overcome these difficulties are the need of the hour to improve the effectiveness and safety of neuroendoscopic surgery [1, 2].

We developed an actuator-driven pulsed water jet device (ADPJ) based on a new concept to achieve both maximal lesion dissection and minimal risk of normal structural damage [6]. Ejected water from the jet penetrates parenchymatous tissue, but vessels and neuronal fibers are preserved because of the differences in elasticity [7]. We reported the usefulness of ADPJ for ventricle dissection with vascular preservation based on tissue selectivity [8, 9]. Although ADPJ has unique characteristics for tissue dissection, the issue of tissue dispersion occurs during dissection.

In this report, we assess the factors associated with dispersion during tissue dissection with ADPJ to reduce the risk of dissemination. At present, there is no established method to evaluate the maneuver-related dispersion of the dissected tissue. Therefore, we propose a simple evaluation method based on spectrophotometry.

## Main text

### Method

#### Actuator-driven pulsed water jet

**Fig. 1:**
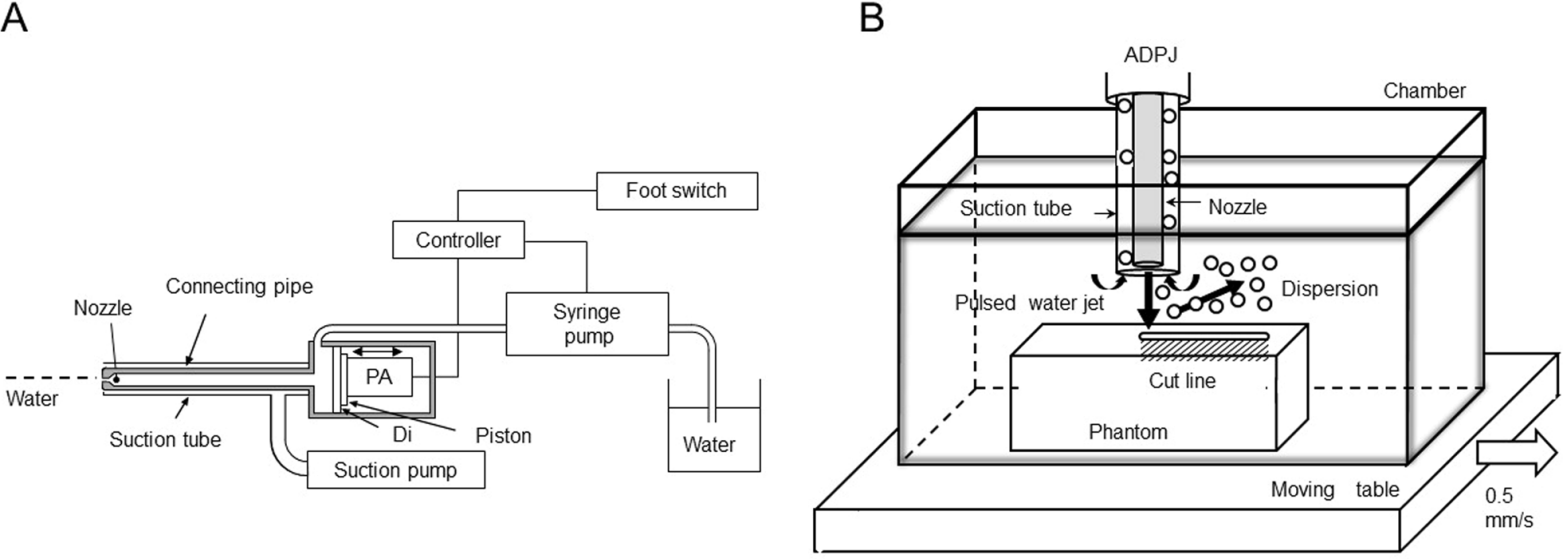
Scheme of the piezo actuator-driven pulsed water jet (ADPJ) system (**A**). The water jet was generated by the movement of the driven piston and ejected from the nozzle tip. *Di* stainless steel diaphragm, *PA* piezo/electric actuator. **B** Scheme of brain phantom dissection in a wet-field condition. Dissected tissue and dispersion were aspirated through the suction tube placed outside the nozzle. Dispersion was recorded by high-speed camera

The basic structure of ADPJ is shown in Fig. 1. Briefly, the piezoelectric actuator unit was attached to the end of the connecting pipe to drive the water (Fig. 1A). The pulsed water was ejected from the nozzle tip. The tip of the nozzle had an inner diameter of 0.15 mm. The suction tube was set outside the connection pipe to remove excess water and splash. The detailed mechanism and device structure are described elsewhere [6].

and then placed in the test chamber filled with 400 mL of the distilled water solution

#### Brain phantoms

Experimental brain phantoms were constructed with gelatin (BCN300S, Nitta Gelatin Co. Ltd., Osaka, Japan) at a concentration of 5% w/v [gelatin weight (g)/total solution volume (mL)]. To assess the dispersion of the debris, the brain phantom was stained with 0.82 weight percent (wt%) methylene blue solution (Japan Pet Design Co. Ltd., Tokyo, Japan). The ratio of methylene blue to the total amount of gelatin was 0.123 wt% gelatin. The methylene blue solution was stirred and dissolved in warm distilled water at 65 °C and then cooled to 4 °C for 10 h for solidification.

#### Brain phantom dissection in wet-field condition

The brain phantom was removed from the storage buffer just before the experiments; 5 mm sections were trimmed off the surface to ensure an equal gelatin surface and then placed in the test chamber filled with 400 mL of the distilled water solution (Fig. 1B). The chamber containing the brain phantom was placed on a solid stage (EZSM 3E020K, Oriental Motor Co. Ltd., Tokyo, Japan) that moved horizontally at 0.5 mm/s. The ADPJ nozzle was set perpendicularly against the phantom surface. A pulsed water jet was ejected toward the brain phantom in the water chamber for 100 s (Fig. 1B). A high-speed camera (NX4S2, IDT Japan Co. Ltd., Tokyo, Japan) was set in front of the nozzle and operated at a rate of 5000 frames/s to observe the dispersion. The dissected brain phantom and the solution were used for further analyses.

#### Factors associated with dispersion

The relationship between the amount of dispersion and the following factors was analyzed: input voltage of the actuator, suction rate, and the distance between the nozzle and the target (standoff distance). The input voltage of the actuator ranged from 20 to 80 V. The suction rate ranged from 0 to 200 mL/min. The standoff distance ranged from 1 to 2.5 mm. The basic operating conditions are input voltage 60 V, suction rate 200 mL/min, flow rate 7.6 mL/min, standoff distance 1.5 mm, and frequency 400 Hz [6, 10]. No other condition was changed during the investigation of a parameter. All experiments were repeated 10 times for each parameter.

#### Methylene blue absorbance

Methylene blue is a near-infrared dye, with a maximum absorbance between 600 and 1000 nm. All absorbance values were measured using an optical spectrophotometer (ASV11D, AS ONE Co. Ltd., Osaka, Japan) [11–13]. The methylene blue-stained solution was poured into a clear cuvette, and absorbance at 664 nm was measured. The measurements were repeated three times for each sample, and the mean was determined.

#### Correlation between methylene blue absorbance and gelatin concentration

**Fig. 2:**
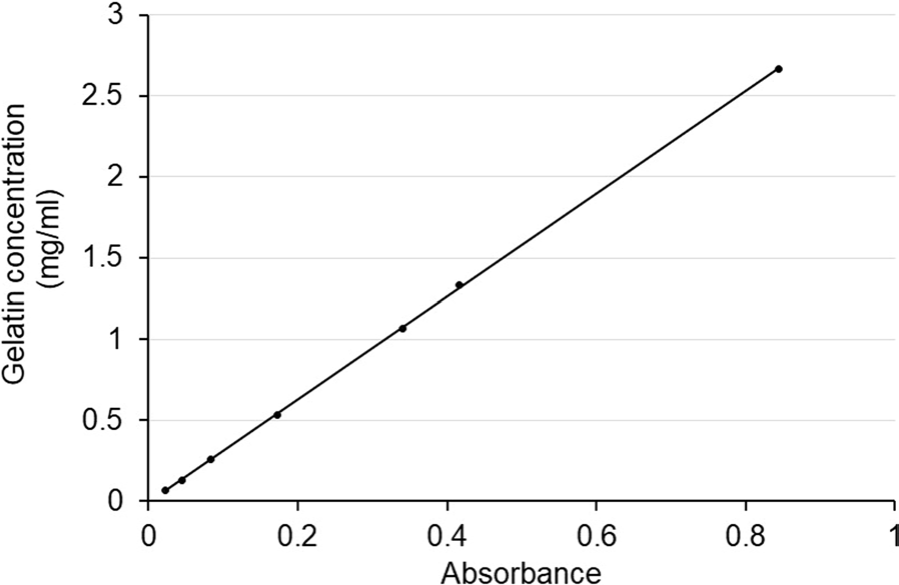
Reference standard curve showing clear proportional correlation between absorbance and concentration

To establish the relationship between the absorbance and the amount of dispersed gelatin, a reference standard curve was constructed. Seven pieces of the blue-stained gelatin, ranging from 20 to 400 mg, were precisely weighed using a laboratory balance (Fx-300i, A&D Co. Ltd., Tokyo, Japan) and dissolved in warmed water (100 mL, 65 °C). The absorbance at 664 nm was measured six times for each solution using a spectrophotometer. A clear proportional correlation was noted between absorbance and concentration, which indicated that measuring the absorbance of the solution can help in evaluating dispersion (Fig. 2).

#### Measurement of dispersion after ADPJ dissection

After dissection by ADPJ, the brain phantom was removed. Subsequently, the solution containing the dispersed gelatin was stirred and warmed to 65 °C for 10 min to dissolve the dispersed gelatin and equalize the concentration. The absorbance of the solution was measured spectrophotometrically, and the total dispersion was calculated using the standard curve described above.

#### Statistical analysis

Pearson product–moment correlation coefficient was calculated using JMP Pro 16 software (SAS Institute Inc., Cary, NC, USA). Statistical significance was set at a p < 0.05. In the correlation test, r showed the correlation between the x- and y-axis. The absolute value of r (|r|) denoted the strength of the correlation, and |r|> 0.7 was defined as a strong correlation. All values were expressed as mean ± standard deviation.

### Results

#### Dispersion and input voltage

**Fig. 3:**
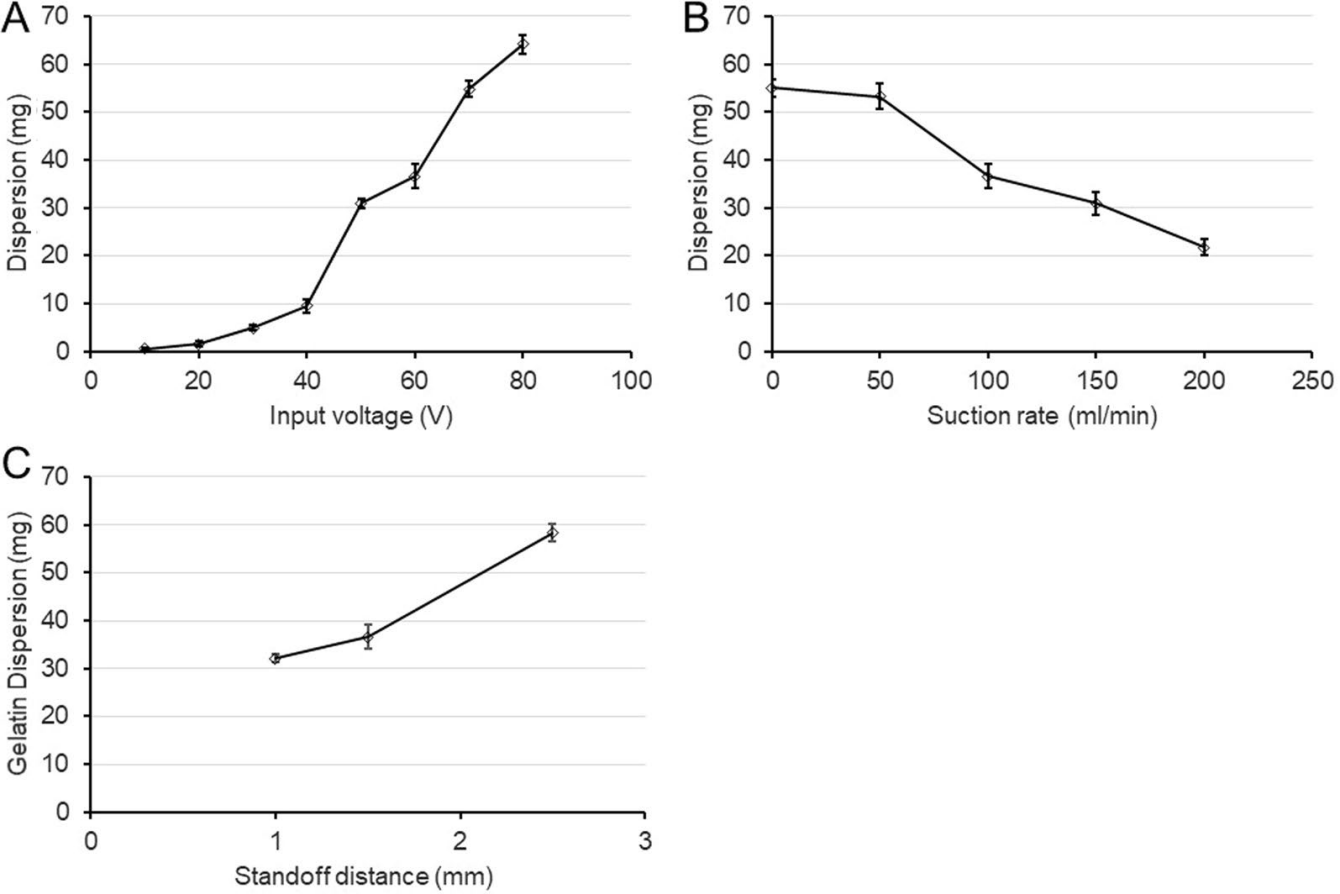
**A** Graph showing the relationship between the input voltage and the amount of dispersion. **B** Graph showing the relationship between the suction rate and the amount of dispersion. **C** Graph showing the relationship between the standoff distance between the nozzle tip and the target and the amount of dispersion

To evaluate the relationship between dispersion and ADPJ input voltage, we examined different voltages, i.e., 20, 40, 60, and 80 V. Other parameters remained the same as the basic operating conditions. With the setting of 20 V, a small amount of dispersion was detected. The amount of dispersion increased as the input voltage was increased (r = 0.9534, p < 0.05) (Fig. 3A).

#### Dispersion amount and suction rate

To assess the relationship between dispersion and ADPJ suction rate, we analyzed different suction rates, i.e., 0, 50, 100, and 200 mL/min. Other parameters remained the same as the basic operating conditions. Without suction, the dispersion was maximum and, with a suction rate of 50 mL/min, the dispersion could not be controlled well. The amount of dispersion decreased with the increase in suction rate (r = −0.8683, p < 0.05) (Fig. 3B).

#### Dispersion and standoff distance

To investigate the relationship between dispersion and ADPJ standoff distance, we checked the different standoff distances, i.e., 1, 1.5, and 2.5 mm. Other parameters remained as per the basic operating conditions. When the standoff distance was closer than 1 mm, gelatin surface was distorted and damaged due to suction force. suction injury of the gelatin block itself occurred. The greater the distance from the target, the higher was the dispersion (r = 0.8853, p < 0.05) (Fig. 3C).

### Discussion

Tremendous efforts have been taken to develop innovative devices that can improve surgical outcomes. New devices are designed based on unique concepts. Device safety and efficacy should be confirmed in an in vitro setting before applying it in an in vivo setting. Since improvement of the dissection profile is one of the most focused issues in the development of new dissection devices, the risk of minute tissue dispersion has not been well studied. Furthermore, there is no established method to evaluate dispersion. This study proposed and confirmed a simple quantitative method to assess tissue dispersion during surgical dissection in a wet field condition. According to this indicator, we assessed the factors associated with tissue dispersion.

We have been engaged in the development of new devices for more than 20 years. ADPJ is one of the most promising dissection devices and has achieved maximal resection with minimal risk of normal structural damage. In the process of in vivo experiments, we detected dispersing debris. Nakayashiki et al. estimated dispersion during dissection maneuvers by measuring the volume of splashed dots in two dimensions [14]. Although this evaluation method is unique and reliable, its application is limited to dry-field dissections. Moreover, small pieces of tissue might be dispersed in three dimensions during surgery.

To overcome such limitations in the evaluation of dispersion, we employed the technique used in basic research. In these fields, accurate measurement of the concentration of certain substances is quite important, but direct measurement is not always available. Thus, indirect methods are widely used. In our study, we dissected a gelatin-based brain phantom using ADPJ; hence, it was difficult to directly quantify the amount of dispersed gelatin in an in vitro setting. Thus, the brain phantom matrix was stained with methylene blue and, by measuring its absorbance, the concentration of the matrix was indirectly inferred.

This study revealed that both dissection force and suction rate are important factors in dispersion. Unsurprisingly, the brain phantoms were dissected more deeply with the use of higher input voltage and greater dispersion. Moreover, a higher suction rate was associated with less dispersion. To achieve effective dissection, adequate dissection force is necessary, and mickle dispersion is inevitable; therefore, strong aspiration is required. Our results suggest that an appropriate standoff distance between the nozzle tip and the target is crucial since a short distance may injure the tissue, while a long distance might increase the risk of dispersion.

## Conclusions

We have described a simple method for evaluating tissue dispersion during surgical maneuvers, which has not been previously established. For the development of new dissection devices, dispersion control is one of the key issues to alleviate the risk of dissemination. Appropriate suction systems can effectively avoid surgical maneuver-related dissemination even with valid dissection devices, such as cavitron ultrasonic surgical aspirator (CUSA) and ADPJ.

## Limitations

The present study has several limitations. First, the surface of the gelatin brain phantom can be dissolved in the solution without dissection during the experiment. A control phantom without ADPJ dissection was immersed in the solution for the same duration as the dissection experiment, and spectrophotometry revealed that elution was very low. Hence, no special consideration was required (data not shown). Second, the suction rate was higher than the ADPJ flow rate, so the volume of the solution in the chamber decreased during the experiment. Thus, we chose 100 s as the duration of dissection to avoid drying up of the solution. In a clinical setting, alteration of intracranial pressure by the excessive drainage of cerebrospinal fluid should be taken into consideration. Third, there might be measurement errors associated with spectrophotometry. Our method did not amplify the signals; hence, the absorbance value was not high with minute deviations, which resulted in reliable results. In fact, the standard curve showed a good correlation between the gelatin concentration and the methylene blue absorbance (Fig. 2). Fourth, although we showed that higher input voltage was associated with increased dispersion, the dissection efficacy was not assessed. In a clinical setting, the maximal dissection of the target lesion is the primary purpose of the surgery. To achieve both effective dissection and a low dispersion rate, further optimization of the ADPJ condition is required. Finally, our method to evaluate dispersion was confirmed by ADPJ only, and there is no comparable data with other dissection devices such as CUSA. The setting condition is completely different among devices, and the results cannot be compared directly. Collecting more data and confirming the findings are planned in the future to strengthen the reliability.

## Data Availability

The datasets used and/or analyzed during the current study are available from the corresponding author upon reasonable request.

## Abbreviations

ADPJ: Actuator-driven pulsed water jet
CUSA: Cavitron ultrasonic surgical aspirator
