# Correction to: Methodological assessment of the reduction of dissemination risk and quantification of debris dispersion during dissection with a surgical aspirator

**DOI:** 10.1186/s13104-022-06167-0

**Published:** 2022-08-15

**Authors:** Sosuke Kageyama, Atsuhiro Nakagawa, Tomohiro Kawaguchi, Kiyonobu Ohtani, Toshiki Endo, Manabu Kyan, Tetsuya Kusunoki, Yoshiteru Shimoda, Shin-Ichiro Osawa, Masayuki Kanamori, Kuniyasu Niizuma, Teiji Tominaga

**Affiliations:** 1 Department of Neurosurgery, Tohoku University Graduate School of Medicine, 1-1, Seiryo-machi, Aoba-ku, Sendai, Miyagi 980-8574 Japan; 2 Department of Biodesign, Clinical Research, Innovation, Education Center, Tohoku University Hospital, Sendai, Miyagi Japan; 3 Department of Neurosurgery, Kohnan Hospital, Sendai, Miyagi Japan; 4 Institute of Fluid Science, Tohoku University, Sendai, Miyagi Japan; 5 Department of Neurosurgical Engineering and Translational Neuroscience, Tohoku University Graduate School of Medicine, Sendai, Miyagi Japan

## Correction to: BMC Research Notes (2022) 15:85 https://doi.org/10.1186/s13104-022-05947-y

Following the publication of the original article [1], it was brought to our attention that the penultimate author's given and family names were unfortunately interchanged.

The name of Prof. Niizuma Kuniyasu should be corrected to Kuniyasu Niizuma, where "Kuniyasu" is the given name and "Niizuma" is the family name.

The correct name is included both in the author list of this Correction and in that of the original article, which has already been revised.
